# X-ray–induced acoustic computed tomography: 3D X-ray absorption imaging from a single view

**DOI:** 10.1126/sciadv.ads1584

**Published:** 2024-12-06

**Authors:** Siqi Wang, Prabodh Kumar Pandey, Gerald Lee, Rick J. P. van Bergen, Leshan Sun, Yifei Xu, Liangzhong) Xiang

**Affiliations:** ^1^ Department of Biomedical Engineering, University of California, Irvine, Irvine, USA.; ^2^ Department of Radiological Sciences, University of California, Irvine, Irvine, USA.; ^3^ Beckman Laser Institute and Medical Clinic, University of California, Irvine, Irvine, USA.

## Abstract

Computed tomography (CT) scanners are essential for modern imaging but require around 600 projections from various angles. We present x-ray–induced acoustic computed tomography (XACT), a method that uses radiation-induced acoustic waves for three-dimensional (3D) x-ray imaging. These spherical acoustic waves travel through tissue at 1.5 × 10^3^ meters per second, much slower than x-rays, allowing ultrasound detectors to capture them and generate 3D images without mechanical scanning. We validate this theory by performing 3D numerical reconstructions of a human breast from a single x-ray projection and experimentally determining 3D structures of objects at different depths. Achieving resolutions of 0.4 millimeters in the *XZ* plane and 3.5 millimeters in the *XY* plane at a depth of 16 millimeters, XACT demonstrates the ability to produce 3D images from one x-ray projection, reducing radiation exposure and enabling gantry-free imaging. XACT shows great promise for biomedical and nondestructive testing applications, potentially replacing conventional CT.

## Teaser

X-ray–induced acoustic computed tomography is a 3D CT imaging technique using only a single projection.

## INTRODUCTION

The advent of three-dimensional (3D) x-ray computed tomography (CT) has advanced our comprehension of intricate structural anatomies in both biological and nonbiological objects (*1*–*3*), widely used across various domains including industrial metrology (*4*, *5*), materials science (*6*, *7*), and biomedical research (*8*–*13*). In the realm of medical diagnostics, CT scanners conduct more than 80 million scans annually in the United States alone (*14*) and serve as indispensable tools for diagnosing acute and chronic conditions. Despite their widespread use, the fundamental principles of traditional CT imaging, as well as the size and weight of CT scanners, have remained largely unchanged over the past half-century (*15*), hindering their accessibility in out-of-hospital settings like in space and other resource-constrained environments (*16*).

**Fig. 1.**
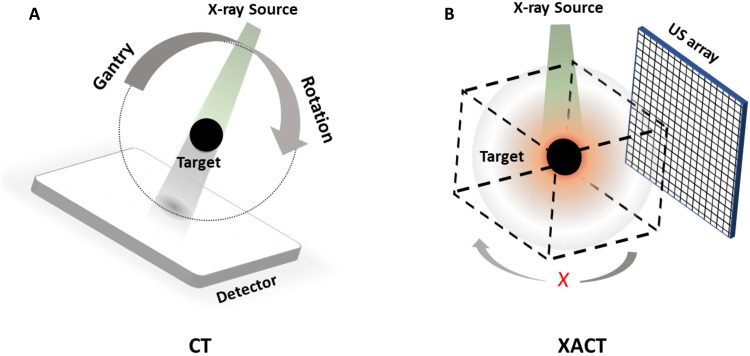
Diagram of the 3D XACT imaging. (**A**) Traditional CT: Acquiring CT images involves numerous projections and the rotation of the gantry to compile a 3D image. (**B**) XACT Method: No gantry rotation is needed for reconstructing a 3D image. In this approach, each element of the ultrasound (US) transducer array receives propagated acoustic waves containing time-of-flight information, which establishes depth in a time-encoded dimension. Using an ultrasound array enables the realization of 3D imaging with just a single x-ray projection.

Conventional x-ray imaging relies on acquiring hundreds of projections to generate a comprehensive 3D image, often necessitating the use of a mechanical gantry for rotation, as depicted in Fig. 1A (*1*, *17*). Researchers are developing imaging approaches for 3D reconstruction without rotating the sample or detectors, such as ankylography in the soft x-ray range (around 6203.49 eV) (*18*, *19*). Yet, no existing method allows for 3D x-ray absorption imaging without gantry rotation in the higher (tens of kilo–electron volts) energy range. Our proposed method, x-ray–induced acoustic computed tomography (XACT) (*20*–*25*), fulfills this need. Instead of relying on projection or mechanical rotation, XACT uses ultrashort x-ray pulses (<μs) to initiate the x-ray–induced acoustic (XA) effect, generating ultrasonic waves (*26*, *27*). These spherical x-ray–induced acoustic waves propagate in all directions from their source and, because of the speed of ultrasound being approximately five orders of magnitude slower than the speed of an x-ray, are easily detected as ultrasound and used for 3D image reconstruction (*21*, *28*). Figure 1B illustrates the concept of 3D XACT using a planar ultrasound transducer array. By recording the time of arrival of acoustic waves, depth information is calculated, allowing the capture of the third dimension in the 3D image.

Despite initial interest in x-ray–induced acoustics dating back to the 1980s (*29*, *30*) and the early 1990s (*31*), it was not until 2013 that substantial advancements in ultrasound transducers and pulsed x-ray sources reignited great interest in the field (*32*). These technological breakthroughs paved the way for the first demonstration of x-ray–induced acoustic tomographic imaging, achieved through mechanical scanning coupled with a linear accelerator (*32*). Subsequent studies showcased the feasibility of obtaining 2D reconstructions of the target medium using a 128-element ring array without the need for mechanical scanning (*33*, *34*). In 2020, Lee *et al.* (*35*) expanded on this by demonstrating 3D reconstructions using a curved linear array and rotating a wire target for 180 steps. However, there remains theoretical potential for achieving 3D reconstructions without any rotational or mechanical scanning of either the x-ray source or the ultrasound detectors. This could be achieved by capturing the 3D spherical x-ray acoustic wave using matrix transducer arrays as described above. Proving this theoretical approach holds promise for further advancing x-ray–induced acoustic tomographic imaging techniques.

Here, we use a 2D ultrasound array to capture 3D x-ray acoustic waves, allowing us to surpass the traditional x-ray absorption imaging limit and reconstruct the 3D x-ray absorption distribution from a single view. We have validated this concept through both computer simulations and experiments, presenting the experimental demonstration in two stages: assessing the depth sensing capability of the 3D XACT system and showcasing the 3D rendering capability of the XACT system. In addition, we demonstrate the 3D imaging potential on a biological sample. This technology plays a pivotal role in enabling portable CT by eliminating the need for gantry rotation, which holds promise for serving rural areas (*36*) and low resource settings (*37*) worldwide. Furthermore, it has notable implications for saving lives on the battlefield (*38*) and potentially facilitating the deployment of CT machines in space stations and other space travel endeavors (*39*).

## RESULTS

### 3D XACT imaging system

We have developed a 3D XACT imaging system consisting of two main components: x-ray–induced acoustic (XA) signal generation and XA signal detection system. For signal generation, we use an ultrashort pulsed x-ray source emitting x-rays with a duration of 50 ns at a repetition rate of 10 Hz and a voltage of up to 150 kilovolt peaks (XR200, Golden Engineering, IN, USA). To capture the XA signal, a 2D matrix ultrasound array with a center frequency of 1 MHz and a bandwidth of 60% [nondestructive testing (NDT) probe, Doppler Co. Limited, Guangzhou, China] is used, followed by signal amplification and acquisition using our dedicated electronic system. Subsequently, the back-projection algorithm is used for 3D XACT image reconstruction (*40*), enabling the generation of 3D images from the captured XA signals, providing comprehensive insights into the internal structures of the imaged objects.

### Numerical simulations of a digital human breast phantom

**Fig. 2.**
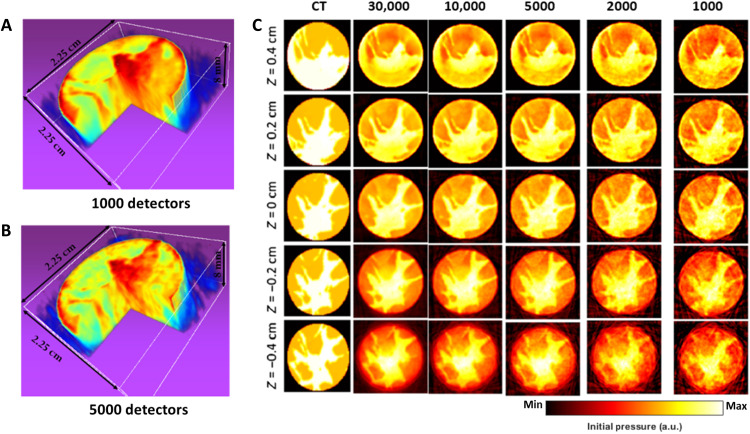
Determination of 3D structure of a simulated human breast with XACT from single x-ray projection. (**A** and **B**) 3D XACT reconstructed volumes of the digital phantom, derived from human breast CT slices, using different detectors. These reconstructions correspond closely with the ground truth of the breast phantom CT images. (**C**) Slices from the 3D XACT simulated reconstruction at various depths, compared with breast CT slices that have been segmented according to three different skin types. The notation “*Z* = 0 cm” indicates the height’s midpoint in the phantom. a.u., arbitrary units.

To verify our theoretical analysis, we executed numerical simulations for the 3D structure determination of a digital human breast phantom using XACT imaging from a single projection. The 3D digital breast phantom was generated from 2D CT slices, rendering a comprehensive model. We assigned distinct acoustic thermoelastic properties to different tissue types—skin, adipose, and glandular—and factored these into the calculations for initial acoustic pressure generation upon photon interaction. Simulation of acoustic wave generation and propagation, starting from the initial pressure and culminating in detection by ultrasound transducer arrays, was conducted using the *k*-space pseudospectral method within MATLAB, used with the k-Wave toolbox (*41*). We assessed the x-ray energy deposition within the breast tissues according to Beer’s law (*6*, *42*). The combination of the x-ray energy deposition map and the Gruneisen parameter map—derived from segmented breast tissues based on their thermal and acoustic properties—served as the source of pressure (*43*). This pressure source was centrally located within a 420 by 420 by 420 grid, featuring a 0.2-mm grid size. We selected a hemispherical surface to represent the detector grid, functioning as a cup array (detailed in note S1). We reconstructed the pressure source using the universal back-projection algorithm (*40*), focusing on a 2.25 cm by 2.25 cm by 0.8 cm cuboidal region within the center of the spherical detection surface. The results of the 3D XACT volumetric reconstruction, using both 1000 and 5000 point detectors, are presented in Fig. 2 (A and B), respectively. For reference, the first column of Fig. 2C shows stacked 3D CT slices from depths of −0.4 to 0.4 cm, serving as the ground truth (left column). The absorption distribution in the reconstructed XACT image of the human breast (right column), obtained from a single projection, closely aligns with that of the actual breast CT images, demonstrating the effectiveness of our XACT approach.

### Imaging multiple objects at different depths

**Fig. 3.**
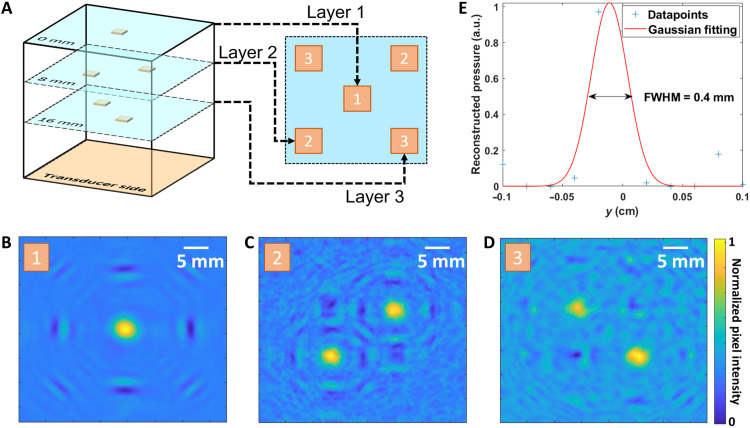
3D structure determination with XACT of multiple objects at different imaging depths. (**A**) Experimental setup schematic of the three-layer embedded lead dots; (**B** to **D**) 3D XACT lateral plane reconstructions of the layered phantom at different depths (0, 8, and 16 mm). (**E**) The point spread function analysis shows that the imaging resolution is about 0.4 mm in the *XY* plane and 1 mm in the *XZ* plane (fig. S2). It slightly degraded with the increase of the imaging depths. FWHM, full width at half maximum.

To illustrate the depth sensing capabilities of the 3D XACT system, we embedded five lead dots, each ^1^/_16_″ (1.59 mm) thick and with a 4 mm by 4 mm surface area, into an agar-based model at three distinct depth levels. Specifically, as depicted in Fig. 3A, we placed one at 0 mm, two diagonally positioned at 8 mm, and another two diagonally positioned lead dots in alternating corners at 16 mm (refer to photos in note S2). In addition, we labeled each embedded lead dot layer in Fig. 3A with letters corresponding to the subfigures (Fig. 3, B to D) of the reconstructions to clarify the link between the actual positions of the targets and the reconstructed images. Using a single projection from a 50-ns pulsed x-ray source located beneath the agar model, the 3D XACT system successfully reconstructed the three layers of embedded lead dots. Figure 3 (B to D) displays the reconstructed XACT images as cross-sectional slices along the *XY* plane at *z*-axis depths of 0, 8, and 16 mm, respectively. Specifically, Fig. 3B presents a clear reconstruction of the central lead dot at the surface layer with optimal image contrast, while Fig. 3 (C and D) reveals the diagonally arranged lead dots at depths of 8 and 16 mm, showing increased background noise with deeper imaging.

The imaging resolution (Fig. 3E) was also evaluated and found to be approximately 3.5 mm in the *XY* plane and 0.4 mm in the *XZ* plane (note S6). These experimental outcomes demonstrate that the XACT system, with a single x-ray projection, can identify targets along the x-ray path, an achievement beyond the capabilities of standard x-ray radiography.

### 3D imaging rendering

**Fig. 4.**
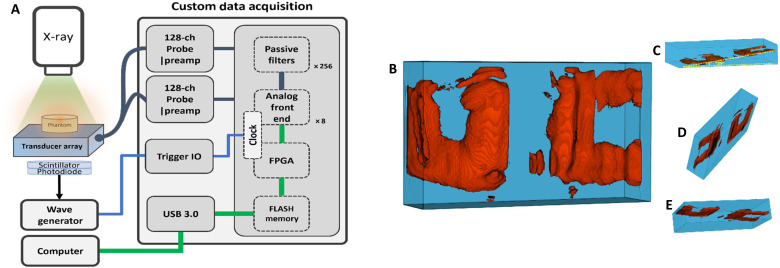
Illustration of XACT imaging for determining 3D structures. (**A**) Schematic of the 3D XACT imaging system, equipped with a 2D matrix ultrasound array. ch, channel; IO, input and output; USB, Universal Serial Bus; FPGA, Field-Programmable Gate Array. (**B**) Front view of the 3D back-projection reconstruction featuring the UC logo within the phantom. (**C** to **E**) Side views of the 3D XACT reconstructed image. The yellow dashed line denotes the angle of inclination (25°) between the UC logo and the base of the agar phantom.

Beyond showcasing the depth sensing capabilities of the 3D XACT system, we also carried out a 3D imaging rendering test to affirm the concept of 3D reconstruction with single projection. In this test, a “UC”-shaped lead logo was positioned within a water-based agar phantom at a 15° angle (as depicted in note S3). The phantom, equipped with a 3D-printed holder for ultrasound transducer array and an x-ray scintillator, was angled at 25° in relation to the transducer’s detecting surface. This orientation ensured that the plane of the UC logo remained perpendicular to the incident x-ray beam, while the scintillator directly faced the x-ray source’s exit window. The lead-embedded phantom was subjected to a single x-ray beam projection. We used bandpass filtering and a back-projection reconstruction algorithm within MATLAB to generate the raw 3D reconstruction matrix. From this, the region of interest containing the UC logo was segmented from the total reconstructed volume (note S4). The 3D-rendered image of the UC lead logo is displayed in Fig. 4B, as shown with a 25° rightward rotation for enhanced 3D viewing (see movie S1). Moreover, a side perspective of the 3D rendering, shown in Fig. 4 (C to E), confirms the expected 15° tilt angle from the experimental setup. It is noteworthy that the center of the UC logo appears more clearly resolved than the logo’s edges within the rendering. This discrepancy is attributed to the uneven distribution of x-ray exposure across the target.

### 3D imaging of biological sample

**Fig. 5.**
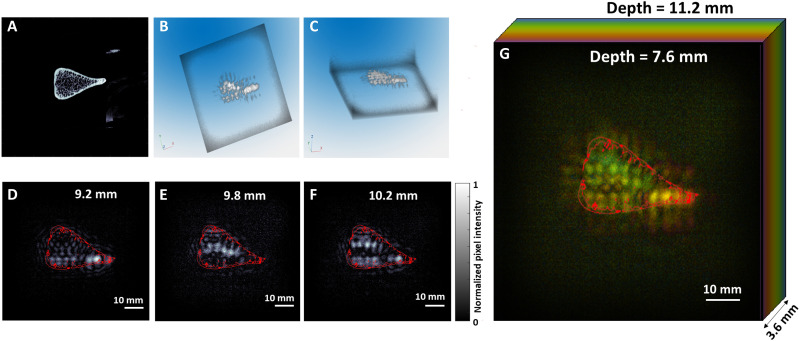
3D XACT imaging experimental study on a biological sample. (**A**) A microCT section scan of a bone sample, used as the reference standard. (**B** and **C**) 3D XACT volumetric reconstructions derived from the study of the bone sample. (**D** to **F**) Slices of the volumetric reconstruction at varying imaging depths: 9.2, 9.8, and 10.2 mm, respectively. (**G**) Color-coded maximum intensity projection based on imaging depths ranging from 7.6 to 11.2 mm.

To showcase the potential of 3D XACT for biological studies, we used the technology to image a pig rib bone, using the same planar matrix ultrasound transducer array used in previous experiments. A microCT scan (Fig. 5A) served as the reference standard, providing a baseline for validating the accuracy of the XACT image reconstructions. For enhanced visualization of the system’s 3D capabilities, we segmented three distinct layers from the volumetric data (Fig. 5, D to F), corresponding to depths of 9.2, 9.8, and 10.2 mm. These layers reveal variations in the bone surface and details of its tilt angle. Furthermore, Fig. 5 (B and C) features 3D views of the bone specimen from different perspectives, providing a comprehensive understanding of its structure. To further illustrate the depth sensing capabilities of our system, we created a color-coded maximum projection over 7.6- to 11.2-mm range in Fig. 5G, showcasing the area where the bone was imaged. The reconstructed 3D XACT images effectively displayed the bone’s structure and depth details. This experiment successfully demonstrates XACT’s valuable applications in the field of biomedicine.

## DISCUSSION

In summary, our research demonstrates the feasibility of obtaining 3D x-ray absorption data through XACT imaging with just a single x-ray exposure. The successful execution of depth sensing and 3D imaging trials on various samples underscores the various applications of this imaging method.

One of the key advantages of XACT imaging over conventional CT is that it enables 3D x-ray absorption imaging with a single x-ray projection, whereas typical CT requires hundreds of projections. As demonstrated in our numerical simulations using a digital human breast phantom (Fig. 2), we can image the breast with just one x-ray projection. The required dose is approximately 0.4 mGy, which is only ^1^/_10_ of that for a typical breast CT (*43*). Our recently published work also shows that XACT dose images can be generated from a single x-ray pulse (4 μs) with submilligray sensitivity using a clinical linear accelerator (*44*). Compared to some emerging imaging technologies, such as laser-induced photoacoustic imaging (*45*–*49*), it offers great potential for providing deeper imaging penetration, making it suitable for whole-body imaging in humans.

In our XACT imaging experiments, both the x-ray source and ultrasound detector array used are commercially available and portable, offering great potential for developing a compact device for 3D x-ray absorption imaging. This is a key advantage over traditional CT systems, which are difficult to make portable due to the need for a large gantry and the requirement for the x-ray source and detector to be positioned on opposite sides of the patient. Looking to the future, laser-driven x-ray sources (*50*, *51*), with their intense, ultrashort pulses, high repetition rates, and energy levels ranging from kilo–electron volts to mega–electron volts, could substantially enhance the performance of XACT imaging. This would further solidify XACT’s role in advancing medical imaging with improved precision and resolution.

Despite the encouraging results from our 3D XACT imaging studies, there are opportunities to enhance the system’s imaging performance. Now, the resolution of the XACT system is constrained by the dimensions and frequency bandwidth of the ultrasound elements (*52*). Our experiments, including those involving the “UC logo” and pig rib bone, demonstrate that the system’s limited spatial resolution affects its ability to capture fine details in both nonbiological (Fig. 4) and biological (Fig. 5) samples. The lateral resolution was approximately 3 mm, which corresponds to the size of the ultrasound elements in the transducer array. Using an array with smaller pitch size could potentially improve the imaging resolution in the *XY* plane. The axial resolution, calculated as *R* = 0.88 ν_S_/*f*_max_ (*27*), where the frequency bandwidth *f*_max_ = 1.3 MHz, was approximately 1 mm. Implementing a higher-frequency transducer array could improve axial resolution. For instance, a 5-MHz center frequency ultrasound array with a 60% bandwidth would provide a resolution of 0.138 mm (*34*), surpassing the resolution of most current CT machines. In addition, when designing the XACT imaging system, it is crucial to consider and satisfy the spatial Nyquist sampling criterion to strike a balance between the number of transducers and achieving an adequate field of view for XACT imaging (*53*, *54*).

The current dependence on matrix array ultrasound probes presents a “limited view” challenge for reconstructing 3D XACT images. Future advancements in image reconstruction algorithms, such as multiple-view acquisition (*55*, *56*), along with model-based approaches (*57*, *58*) and deep learning methodologies (*59*–*61*), could potentially overcome these limitations.

Another limitation of this work is that we have only conducted experiments on a human-mimicking phantom (Fig. 4) and ex vivo samples of pig rib bone (Fig. 5). In vivo validation is essential for addressing potential challenges such as tissue heterogeneity, blood flow, and movement, which may affect the performance of the XACT imaging system (*62*–*64*). This will be a key focus for future animal experiments. Furthermore, transmission x-ray imaging will be incorporated as prior information, providing anatomical details that can be used to enhance the accuracy of XACT imaging reconstruction.

Ultimately, our findings confirm that XACT can generate 3D structural reconstructions with just one x-ray projection, a process verified through both simulations and experimental practices. This approach of single-projection imaging removes the necessity for complete access or rotation around the target, proposing a substantial leap forward for medical diagnostic imaging and nondestructive evaluation. Particularly in scenarios where rotating the sample or accessing different angles is challenging, this method could serve as a viable alternative to conventional x-ray CT, heralding a new era in absorption-based imaging techniques. The continued evolution of this technique, supported by the wider research community, presents great potential for enhancing medical imaging and nondestructive testing methodologies.

## METHODS

### Experimental setup

In the XACT setup (illustrated in Fig. 4A), the system consists of two main components: (i) an x-ray source that generates acoustic signals and (ii) an apparatus to detect these acoustic waves. The setup features a pulsed x-ray generator (XR200, Golden Engineering, IN, USA) positioned above the target, which emits x-ray photons downward. Although capable of producing x-rays at a frequency of 10 Hz, we used just a single 50-ns pulse for each experiment in both the three-layer depth sensing and the 3D XACT volumetric reconstructions. The output dose from the x-ray source is approximately 2.6 mR per pulse at 12 inches (30.48 cm) from the source with a 40° projection angle. For acoustic wave detection, the system uses a 256-element matrix ultrasound array (NDT probe, Doppler Co. Limited, Guangzhou, China), designed to pick up x-ray–induced acoustic waves to facilitate 3D imaging. In addition, the detection apparatus includes a photodiode and a scintillator for identifying proton pulses, which act as trigger signals for the imaging process. A cerium-doped lutetium oxyorthosilicate (Ce: Lu_2_SiO_5_) crystal (MTI Corporation, CA, USA) positioned below the x-ray source functioned as a scintillator, converting x-ray photons to blue light. This light was subsequently transformed into electric trigger pulses by an enhanced photodetector (APD410C, Thorlabs, NJ, USA). These pulses were transmitted to a wave generator (33522B, Keysight, CA, USA), which subsequently provided the Legion Analog-to-Digital Converter (ADC) Data Acquisition (DAQ) system with 4-V rectangular pulses to ensure precise timing during the experiments. These triggers are converted into digital signals by a function generator (Keysight, USA), and these data are then relayed to a computer for subsequent postprocessing and image reconstruction. The system’s amplifiers provide up to 91 dB of signal amplification, enhancing the detection of acoustic signals for clearer imaging results. This XACT imaging that uses a matrix array allows for 3D imaging without mechanical scanning.

### Imaging protocols

In the experiments depicted in Figs. 3 to 5, we used target samples embedded within 3% agar phantoms (Bacto, Becton, Dickinson and Company, NJ, USA) as the imaging subjects. The ultrasound waves induced by x-ray exposure in these samples were detected using a 256-element planar ultrasound transducer array. This array operates at a central frequency of 1 MHz and has a bandwidth of 60%. The signals captured by the array were then processed using a sophisticated 256-channel data acquisition (DAQ) system, the Legion ADC (PhotoSound Technologies Inc., Houston, USA), which includes dual integrated preamplifiers for each 128-channel set. The initial phase involved conditioning the acoustic analog signals, which were then filtered and digitized for further analysis. These digital data were stored temporarily in a memory buffer before being transferred to a workstation for detailed evaluation.

For 3D XACT imaging using a singular x-ray projection angle, settings on the Legion ADC system were adjusted to collect 2000 samples per trigger at a sampling frequency of 40 MHz. This configuration accommodates a sound travel duration of 50 μs, covering a 7.5-cm path of XA wave propagation within the agar, thus ensuring comprehensive coverage of the region of interest surrounding the target phantoms for detailed reconstruction. During postprocessing, the recorded XA signals underwent refinement via a digital lowpass filter with a cutoff frequency of 3 MHz and an attenuation rate of −90 dB. The image reconstruction for these studies was executed using a MATLAB-based program, using a universal back-projection algorithm (*40*) for precise imaging outcomes.

### 3D XACT image reconstruction

The acoustic field propagation following the x-ray energy deposition in material under the stress and thermal confinement and negligible acoustic attenuation is based on (*65*)

$$\frac{\partial^{2}p\left(\vec{r},t\right)}{\partial t^{2}}-c^{2}\nabla^{2}p\left(\vec{r},t\right)=\Gamma H\left(\vec{r}\right)\frac{\partial \delta \left(t\right)}{\partial t}$$ (1)

with $\Gamma \;\left(=v^{2}\beta /C_{p}\right)$ being the Gruneisen parameter, and v,β, and $C_{p}$ being the sound speed, volumetric expansion coefficient, and the specific heat at constant pressure. *H* indicates the x-ray energy deposition, and the initial pressure source (p) is the product of the Gruneisen parameter (Γ) and x-ray energy deposition (*H*). The solution to Eq. 1 is given by (*66*)

$$p\left(\vec{r},t\right)=\frac{\Gamma }{4\pi c}\frac{\partial }{\partial t}\left[\frac{1}{\mathrm{vt}}\int_{S\left(\vec{r\prime },t\right)}H\left(\vec{r}\prime \right){\mathrm{dS}}^{\prime }\left(t\right)\right]$$ (2)

$$|\vec{r}-\vec{r}\prime |=\mathrm{vt}$$

where $S^{\prime }\left(t\right)$ represents a spherical surface centered at a detector (located at $\vec{r}$) with radius vt.

The inverse problem corresponding to the XACT is to obtain a 3D map of x-ray energy deposition $\left[\tilde{H}\left(\vec{r}\right)\right]$ from a set of boundaries XA measurements $p\left({\vec{r}}_{0},t\right)$ made at detectors located at ${\vec{r}}_{0}$ on the detector grid *S*_0_ (note S5). In this work, we use the universal back-projection algorithm for the reconstruction (*40*), which is given by

$${\tilde{p}}_{0}\left(\vec{r}\right)=\Gamma \tilde{H}\left(\vec{r}\right)=\int_{S_{0}}b\;\left({\vec{r}}_{0},t=\frac{|{\vec{r}}_{0}-\vec{r}|}{v}\right)\;\frac{d\Omega }{\Omega }$$ (3)

where the back-projection term $b\;\left({\vec{r}}_{0},t\right)$ = 2 $p\left({\vec{r}}_{0},t\right)-2t\frac{d\;p\left({\vec{r}}_{0},t\right)}{\mathrm{dt}}$. Different sound speeds will be assigned to various materials to account for and mitigate the effects of acoustic inhomogeneity (*62*–*64*).

The x-ray energy deposition map $H\left(\vec{r}\right)$ is a nonlinear function of x-ray absorption coefficient map $\mu_{a}\left(\vec{r}\right)$

$$H\left(\vec{r}\right)=\mu_{a}\left(\vec{r}\right)\Phi \left(\vec{r}\right)$$ (4)

with $\Phi \left(\vec{r}\right)$ being the local x-ray fluence. The nonlinearity arises due to the intrinsic dependence of the fluence $\Phi \left(\vec{r}\right)$ on the $\mu_{a}$ distribution in the domain, expressed by the Beer-Lambert Law

$$\Phi \left(\vec{r}\right)=\Phi_{0}e^{-\int_{l^{\prime }}\mu_{a}(\vec{r}\prime )dl}$$ (5)

where $\Phi_{0}$ is the source fluence and $\int_{l\prime }\mu_{a}\left(\vec{r}\prime \right)\mathrm{dl}\prime$ represents the line integral of absorption coefficient distribution $\mu_{a}\left(\vec{r}\prime \right)$ along the line l′ connecting the source with point $\vec{r}$.

The pixel intensity in the XACT images, reconstructed from captured x-ray–induced acoustic signals, represents the initial acoustic pressure distribution. Therefore, the relative intensity image offers vital information regarding x-ray absorption of the targets. Future research will focus on developing fluence correction algorithms to enable quantitative x-ray absorption coefficient reconstruction from XACT.

## Supplementary Materials

### The PDF file includes:

Supplementary Notes S1 to S6 Figs. S1 to S6 Legend for movie S1
