# Supplementary material for: X-ray–induced acoustic computed tomography: 3D X-ray absorption imaging from a single view
Source: Sci Adv. 2024 Dec 6;10(49):eads1584. doi: 10.1126/sciadv.ads1584 (PMC11627201; doi:10.1126/sciadv.ads1584)

Science Advances
AAAS

Supplementary Materials for

X-ray–induced acoustic computed tomography: 3D X-ray absorption imaging from a single view

Siqi Wang *et al.*

Corresponding author: Shawn (Liangzhong) Xiang, liangzhx@hs.uci.edu

Sci. Adv. **10**, eads1584 (2024)
DOI: 10.1126/sciadv.ads1584

The PDF file includes:

Notes S1 to S6
Figs. S1 to S6
Legend for movie S1

Other Supplementary Material for this manuscript includes the following:

Movie S1

Supplementary Note 1- Numerical study on XACT breast imaging

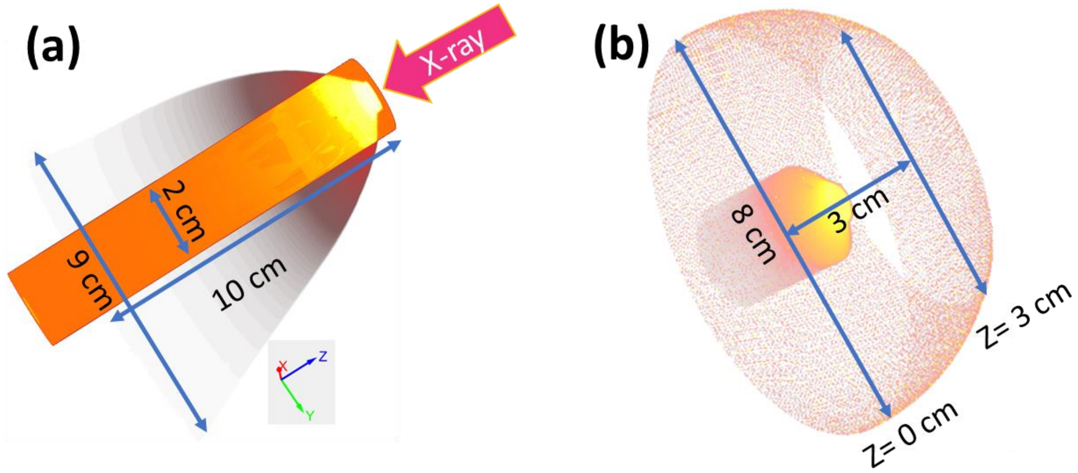

Supplementary Fig. S1: Layout of the X-ray projection and ultrasound detection array used in the simulation study. (a) Illustration of the simulated setup for the X-ray source. (b) Representation of the simulated configuration for the cup-shaped array of ultrasound detectors.

For the 3D XACT simulation studies, a collection of computed tomography (CT) images representing cross-sections of a breast were utilized as the initial pressure sources for simulating acoustic wave propagation. The CT images were segmented into three distinct tissue types: skin, adipose, and glandular tissues, based on their Hounsfield unit values. A total of 487 breast cross-section images were processed and compiled into a three-dimensional digital phantom, as outlined in reference (*43*).

The simulations involved a pulsed cylindrical X-ray beam with a 2 cm diameter targeting the breast model, as depicted in Supplementary **Fig. S1a**. The design for the detector setup was a hemispherical cup array with a 4 cm radius and a 3 cm height, featuring a central opening, as illustrated in Supplementary **Fig. S1b**. The X-ray-induced acoustic signals' propagation was simulated with a 20 MHz sampling rate, and these signals were captured at 30,508 uniformly distributed points across the cup array's surface.

To perform the 3D XACT numerical studies, a series of computed tomography (CT) breast cross section images were obtained to be used as the initial pressure source for the acoustic propagation simulation. To differentiate between different tissue types, the CT slices were individually segmented into three categories: skin, adipose and glandular tissue based on the Hounsfield scale of examined areas. In total, 487 breast cross sections were acquired and volumized into a three-dimensional digital phantom.

Supplementary Note 2- Imaging multiple objects at different depths

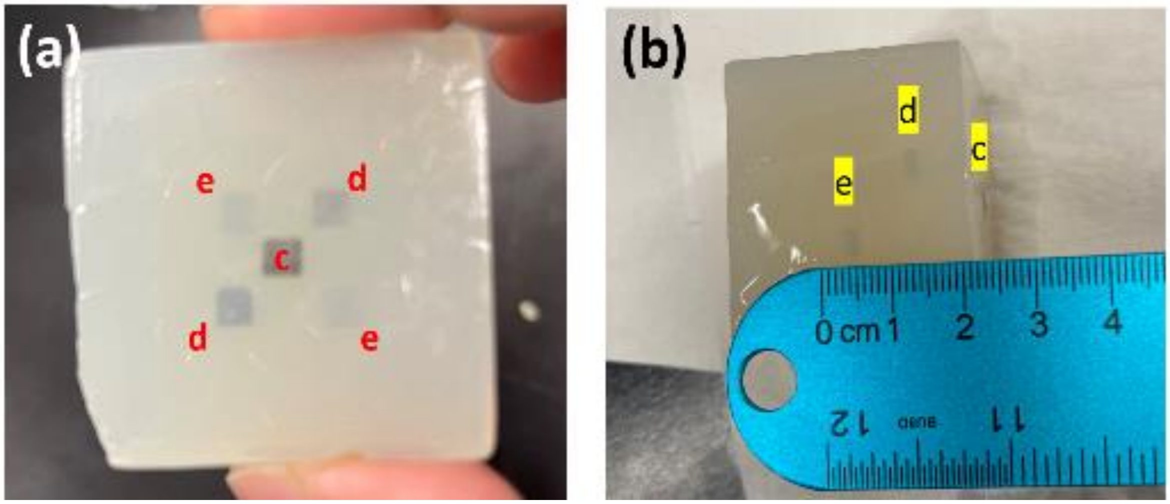

Supplementary Fig. S2: targets placed within a phantom at three different depths. (a) Photo showcasing a top-down perspective of the phantom with three layers of embedded lead dots. (b) Photo displaying a lateral perspective of the phantom containing the three-layer arrangement of embedded lead dots.

Beyond the illustrative representation of the lead dots' arrangement for the tiered 3D XACT showcase, photographic images of the phantom with embedded lead dots are provided, as visible in **Fig. S2a** and **S2b**. Five lead dots were embedded in a phantom, each 1/16” thick and with a 4x4 mm^2 surface area, into an agar-based model at three distinct depth levels.

Supplementary Note 3- Experimental photo

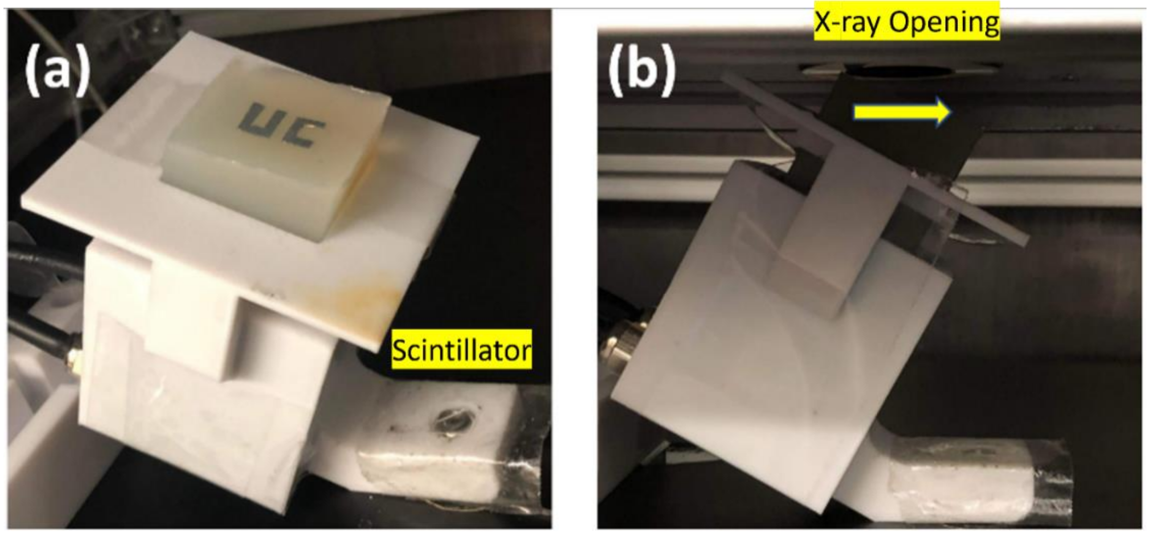

Supplementary Fig. S3: 3D ‘UC’ logo setup. (a) Top view of the 3D printed housing fabricated for the ‘UC’ logo experiment. (b) Final experimental setup for the ‘UC’ logo 3D XACT demonstration.

The arrangement for the 'UC' logo 3D XACT experimental display is shown from various perspectives in **Fig. S3a** and **S3b**. A 3D-printed white casing was constructed to secure the matrix array ultrasound transducer, the 'UC' logo-embedded agar phantom, and the scintillator crystal sensitive to X-ray photons in place. In the course of the experiment, the agar phantom and the matrix array transducer were positioned at a 25-degree angle relative to the surface of the table, while the scintillator was situated directly below the X-ray source.

Supplementary Note 4- Manual segmentation in XACT image

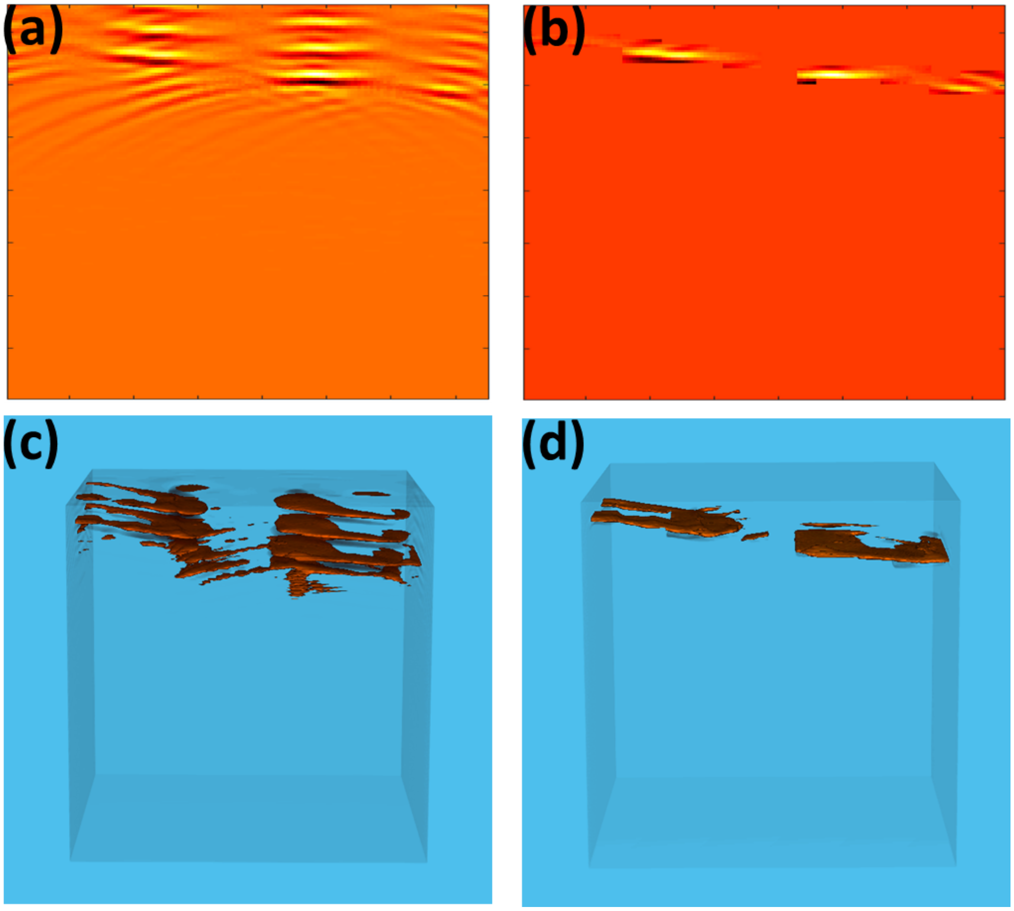

Supplementary Fig. S4: Manual segmentation applied to XACT images utilizing maximum intensity projection techniques (a) The initial maximum intensity projection of the 3D XACT 'UC' logo phantom as reconstructed along the row axis of the matrix array transducer; (b) The manually segmented maximum intensity projection from the 3D XACT 'UC' logo phantom along the same direction; (c) The original 3D volumetric reconstruction of the 'UC' logo phantom aligned with the row direction of the matrix array transducer; (d) The manually segmented 3D volumetric reconstruction of the 'UC' logo phantom following the row direction of the matrix array transducer.

Acoustic impedance mismatches between the lead target and the phantom caused observable echoes in the initial 3D XACT reconstructions, shown in **Fig. S4a** and **Fig. S4 c**. To demonstrate the 3D XACT imaging concept effectively, manual segmentation was applied to improve the 'UC' logo reconstruction, based on its maximum intensity projection along the transducer's row direction. The before and after segmentation effects on the reconstructions are illustrated in **Fig. S4b** and **Fig. S4d**, respectively.

Supplementary Note 5- 3D XACT image reconstruction algorithm

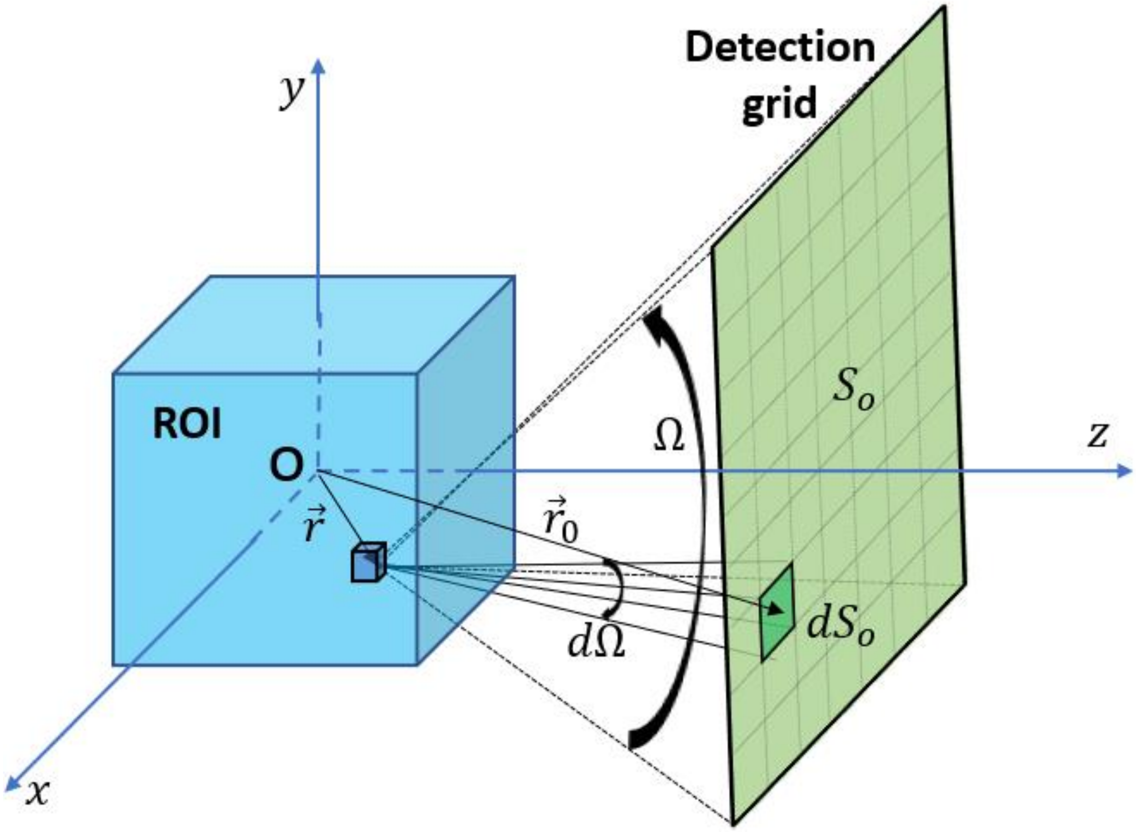

Supplementary Fig. S5: Schematic for UBP based image reconstruction: Image reconstruction is performed voxel by voxel. For every voxel, we evaluate the UBP equation (Eq. (3)) numerically, which in discrete domain is the weighted summation of the contribution terms $b\left(\vec{r}_0, t = \frac{|\vec{r}_0 - \vec{r}|}{v}\right)$ from each transducer elements.

Supplementary Note 6- Imaging resolution test in three dimensions

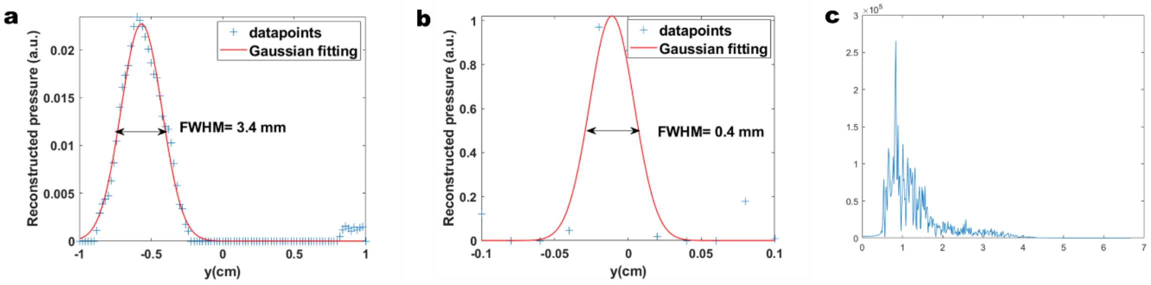

Supplementary Fig. S6: Three-dimensional imaging resolution assessment. (a) The resolution in the X-Y plane, parallel to the matrix array, was measured at 3.4 mm. (b) In the X-Z plane, perpendicular to the transducer surface, the resolution was determined to be 0.4 mm. (c) Frequency spectrum analysis using a 1 MHz central frequency array; the highest detectable frequency ranges between 3 to 4 MHz.

In **Fig. S6**, the XACT result illustrates a small piece of lead imbedded in the phantom. Fig. **S6a and S6b** present the normalized intensity profile along x=0 mm line in **Fig. 4d**, where pixel intensities are represented by dots. The curve depicts the fitted line spread function, with a full-width at half-maximum of 3.4 mm in X-Y plane which close to the element size of the ultrasound array, indicating an approximate lateral spatial resolution of 3.4 mm. The resolution in X-Z plane is about 0.4 mm which is determined by the highest frequency the ultrasound detector can catch which is about 3 to 4 MHz.

Supplementary Movie 1: 3D XACT imaging of “UC logo” target

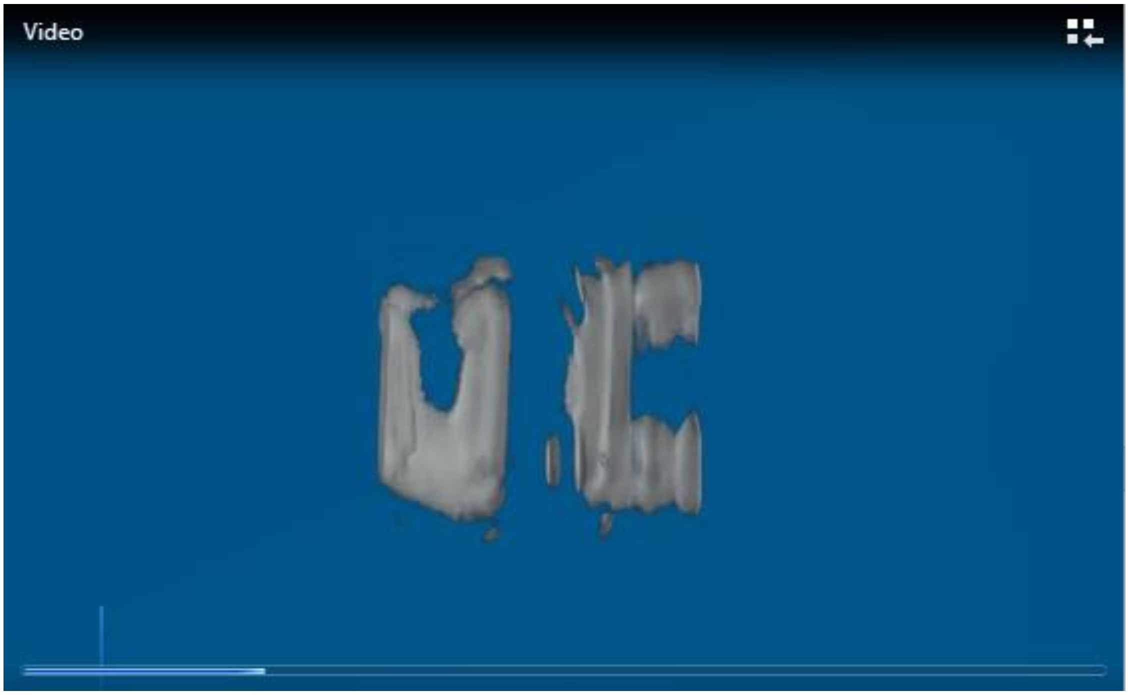

Supplement: Supplementary file 1 — Supplementary Notes S1 to S6 Figs. S1 to S6 Legend for movie S1 [file sciadv.ads1584_sm.pdf]
